# Age-dependent differences in survival of patients with early breast cancer: analysis of SUCCESS A, B, C trials

**DOI:** 10.1007/s00404-025-08255-5

**Published:** 2026-02-19

**Authors:** Stefan Lukac, Davut Dayan, Wolfgang Janni, Brigitte Rack, Visnja Fink, Kristina Veselinovic, Kerstin Pfister, Angelina Fink, Thomas W. P. Friedl, Elena Leinert

**Affiliations:** Department of Obstetrics and Gynecology, University Hospital Ulm, Prittwitzstrasse 43, 89075 Ulm, Germany

**Keywords:** Breast cancer, Age, Elderly, Chemotherapy, Survival

## Abstract

**Purpose:**

Elderly breast cancer (BC) patients are commonly at risk of under-treatment, which can negatively affect their prognosis. Therefore, we analyzed age-related survival differences considering clinico-pathological parameters among patients with early BC.

**Methods:**

8190 BC patients from SUCCESS A, B, and C trials who underwent surgery and adjuvant systemic therapy were analyzed. Tumor and nodal stage, grading, biological subtype, types of surgical and systemic therapies, and other clinico-pathological parameters were compared between age groups ≤ 50 years, 51–65 years, > 65 years (chi-square tests). Breast cancer-free interval (BCFI), breast cancer-specific survival (BCSS), overall survival (OS), invasive disease-free survival (iDFS), and distant disease-free survival (DDFS) were analyzed using univariable and adjusted multivariable Cox regression models. Two-way interactions between age and other clinico-pathological parameters were calculated.

**Results:**

There were significant differences between the age groups concerning almost all parameters analyzed, especially more advanced tumor stages in the elderly group. Univariable analysis showed significant differences between the age groups for all survival parameters (all *p < *0.001). However, after adjustment for other prognostic parameters, an independent significant age effect was found only for iDFS (*p = *0.038), while there was no significant independent age effect on BCFI (*p = *0.286), BCSS (*p = *0.981), OS (*p = *0.131) and DDFS (*p = *0.316).

**Conclusion:**

Although elderly BC patients > 65 years had poorer survival, multivariable analysis suggests this difference is mostly attributable to advanced tumor stages rather than age itself. Effective BC screening and research related to the influence of (biological) age on response to cancer therapies could be the next step to improve understanding the relationship between patient age and BC survival.

## What does this study add to the clinical work below?

**Table:** 

Poor outcomes in elderly patients with early breast cancer are mostly attributable to advanced tumor stages at diagnosis rather than age itself. Treatment decisions should therefore be based on prognostic and predictive factors, not chronological age alone, to avoid under-treatment of elderly patients.	

## Introduction

The incidence of breast cancer (BC) increases significantly after the age of 50, with a median age at first diagnosis of 64 years according to data from the German Cancer Society [1, 2]. Genetic, epigenetic, biological, and environmental factors are discussed as causative factors for the timing of the disease [3, 4]. Older patients are more likely to present with tumors in locally advanced stages but with more favorable prognostic factors than younger women, who are more likely to present with triple-negative and poorly differentiated tumors [5, 6]. Still, older patients with BC are less likely to receive standard guideline-adherent treatment [6, 7]. Some retrospective data show that patients receive chemotherapy and radiotherapy less frequently with increasing age [7, 8]. Consequently, many existing retrospective studies that address age-related prognosis report that under-treatment of older patients is reflected in poorer overall survival [6, 9, 10]. However, recent studies suggest that the impact of age upon BC survival is more complex and the primary influence of age itself on survival remains unclear [11]. Thus, this study investigates the impact of age on various survival parameters in a large cohort of early BC (eBC) patients, who received standardized adjuvant chemotherapy, according to study protocol as part of a clinical trial.

## Material and methods

### Data collection

In the present study, data from a total of 8190 patients with early and locally advanced breast cancer who participated in the adjuvant SUCCESS A (NCT02181101), SUCCESS B (NCT00670878), or SUCCESS C (NCT00847444) trial were analyzed. These studies represent a series of three consecutive clinical trials conducted between 2005 and 2017 on primary intermediate-to-high-risk breast cancer patients. Patient accrual for these three trials spanned from 2005 to 2011 (3754 patients from 2005 to 2007 in SUCCESS A, 793 patients from 2008 to 2011 in SUCCESS B, 3643 patients from 2009 to 2011 in SUCCESS C).

Main inclusion criteria for all of the SUCCESS trials were primary R0-resected epithelial invasive breast cancers (pT1-4, pN0-3, pM0), and—importantly in the context of any analysis regarding age effects—all patients had ECOG (Eastern Cooperative Oncology Group) performance status ≤ 2. All patients received obligatory radiotherapy following breast-conserving surgery and in case of ≥ 4 axillary lymph node metastases. For patients undergoing mastectomy, radiotherapy was also recommended for tumors > 3 cm and in case of 1–3 involved lymph nodes if at least one additional risk factor (multi-centric growth, lymphangiosis carcinomatosa, pectoralis fascia involvement, safety margin of < 5 mm) was present. Adjuvant endocrine therapy for premenopausal and postmenopausal patients with steroid hormone receptor (sHR)-positive tumors (≥ 10% of the cells in the tumor tissue positive for estrogen and/or progesterone receptors) and HER2-targeted therapy for patients with HER2-positive tumors (IHC score 3 + or IHC score 2 + FISH positive) were administered following current guideline standards as defined in the respective study protocol (see Appendix 1 for more details).

The first follow-up was performed 4 weeks after the last course of chemotherapy and 6 weeks after the last administration of radiotherapy. Further controls were conducted every 3 months for the first 3 years and every 6 months during the following 2 years, in accordance to guidelines for breast cancer follow-up.

Three age categories were defined to evaluate the age factor. Patients older than 65 years are commonly referred to as "elderly" and are defined as a separate group in many demographic evaluations; in the following, these patients are referred to as age category C. Female patients between the ages of 51 and 65 (middle age) are assigned to age category B. Age category A includes all female patients up to 50 years of age. This age classification of patients has been used in several studies and correlates with the age-related incidence of breast cancer [12, 13].

The SUCCESS studies were approved by the responsible ethics committees (SUCCESS A: Ludwig Maximilian University Munich, 076/05; SUCCESS B: Ludwig Maximilian University Munich, 395/07; SUCCESS C: Heinrich-Heine University Duesseldorf, MC-LKP-319) and were performed in accordance with both Good Clinical Practice and the Declaration of Helsinki. Written informed consent was obtained from all patients participating in one of the SUCCESS trials.

### Parameters analyzed

#### Clinical parameters

Based on body height and weight at the time of study enrollment, the body mass index (BMI) in kg/m^2^ was calculated and the patient was assigned to the BMI-categories underweight, normal weight, overweight, and obese according to the WHO classification [14].

Menopausal status at the time of study enrollment was categorized as premenopausal or postmenopausal. The type of surgery (breast-conserving, mastectomy, other) was based on clinical standards and was decided individually for each patient. Data on the axillary intervention were not included. As a measure of general physical health condition at the start of chemotherapy, ECOG performance status immediately prior to start of the first chemotherapy cycle was documented.

Patients participating in the SUCCESS trials received three different types of adjuvant chemotherapies:

1. Three cycles of 5-fluorouracil, epirubicin, and cyclophosphamide every 3 weeks (q3w); followed by 3 cycles of docetaxel and gemcitabine d1,8 q3w (FEC-DOC-G); SUCCESS A, B
2. The first 3 cycles were identical but followed by 3 cycles of docetaxel q3w only (FEC-DOC); SUCCESS A, B, C
3. Six cycles of docetaxel and cyclophosphamide q3w (DOC-C); SUCCESS C

To assess possible under-treatment, we documented whether a patient had any dose reduction (yes/no) or any dose delay of more than 6 days due to toxicity (yes/no) during chemotherapy.

Endocrine treatment, HER2-targeted therapy, and the use of radiotherapy were documented and considered as binary (yes/no) categorical variables. However, it has to be noted that endocrine therapy, which was not administered as a study treatment, was not documented consistently for all patients as many patients received their endocrine therapy at their general practitioners or gynecologists practice rather than at the study center. As this information was not always provided to the study center, the documentation of endocrine therapy was incomplete, and patients may have been misclassified as having received no endocrine therapy. HER2-targeted therapy comprised mainly, but not exclusively, treatment with trastuzumab, as trastuzumab was only approved in the adjuvant setting in 2006 after the start of the SUCCESS A study.

#### Pathological parameters

Pathological tumor stage (pT1, pT2, pT3, pT4), nodal status (pN0, pN1, pN2, pN3), and histological grading (G1, G2, G3) were obtained. For sHR status and HER2 status, tumors were classified as positive or negative following the definitions presented above. Tumor classification by histologic type (ductal, lobular, other) was based on the current histopathologic classification.

Biological subtype was defined in the absence of consistent Ki-67 determination in the following manner: Luminal A: sHR-positive, HER2-negative, G1-2; Luminal B: sHR-positive, HER2-negative, G3; HER2 type: sHR-positive/-negative, HER2-positive; triple-negative BC: sHR-negative, HER2-negative.

### Survival

Survival analysis was performed using the following survival endpoints as defined according to the STEEP criteria [15]. To specifically evaluate the effect of age on breast cancer recurrences, we used breast cancer-free interval (BCFI) as endpoint of special interest, with invasive ipsilateral, local, and distant recurrences as well as breast cancer-related deaths as event, while invasive contralateral breast cancer, ipsi- or contralateral DCIS, second primary non-breast invasive cancers, and non-breast cancer-related deaths were excluded. Overall survival (OS) considers all deaths as an event that occurred regardless of the cause. For the calculation of invasive disease-free survival (iDFS), invasive ipsilateral, local or distant disease progression, patient death from all causes, as well as invasive contralateral breast cancer, and second primary non-breast invasive cancers were counted as an event, whereas ipsi- or contralateral DCIS was not. Distant disease-free survival (DDFS) includes the occurrence of distant metastasis or death from any cause as an event. To assess the impact of age on breast cancer-related deaths only, we also included breast cancer-specific survival (BCSS) as commonly used endpoint (even if not specifically defined by STEEP criteria); for the calculation of BCSS, only the death of a patient that was clearly attributable to breast cancer was considered an event.

For all survival analyses reported here, survival was calculated from the date of randomization to the earliest time of occurrence of an event or to the last time that an adequate assessment occurred. If no appropriate event was documented for a patient, she was censored at the time of the last adequate assessment. For the calculation of BCSS and BCFI, patients that died for reasons not related to breast cancer or for unknown reasons were censored at the date of death.

### Statistical analysis

Data were analyzed using IBM SPSS (Statistical Package for the Social Sciences) version 26 (SPSS Inc). All p values reported are two-sided and *α* = 0.05 was used as the significance level throughout; no adjustment was made to the significance level for multiple testing.

Characteristics of the female patients obtained in terms of categorical variables were described using frequency tables, and each was reported in absolute numbers and percentages within each of the three age categories. Metric variables (age and BMI) were reported as medians and ranges.

The associations between age categories and categorical parameters were analyzed by chi-square tests in cross-tabulations, and associations between age categories and metric variables were analyzed using the Kruskal–Wallis H test.

The survival endpoints BCFI, BCSS, OS, iDFS, and DDFS were analyzed based on Kaplan–Meier estimates, illustrated using Kaplan–Meier curves and compared between age categories using a log-rank test. Hazard ratios (HR) with 95% confidence intervals (CI) for the age categories were first calculated using univariable Cox proportional hazard regression models, with the group of female patients > 65 years as the reference category. Then, multivariable Cox regression models were used to analyze the adjusted independent effects of the observed clinicopathologic parameters including age categories on the five survival endpoints. To examine whether there was an effect of age on survival that depended on other clinicopathologic parameters, the two-way interactions between age and each of the other parameters (excluding menopausal status) were examined with a Cox regression model calculated with the main effects of age and the other examined parameter together with the two-way interaction between the two variables.

## Results

The age groups ≤ 50 years, 51 – 65 years, and > 65 years of age comprised 3094 (37.8%), 3703 (45.2%), and 1393 (17.0%) of patients, respectively. While the age group > 65 years included patients up to 86 years, it is important to note that only 37 of the 1393 patients in this age group were older than 75 years. The detailed descriptive statistics of patient characteristics according to age category together with the results of the tests for association between age category and clinico-pathological parameters can be found in Table 1. Importantly, patients > 65 years were more often overweight or obese, had higher tumor and nodal stages, had more often a mastectomy, and had more often dose reductions or dose delays > 6 days due to toxicity compared to younger patients (see Table 1).

**Table 1:** Patient characteristics (clinico-pathological parameters) of the patients recruited in the SUCCESS trials according to age category

		Age categories			*p* value^§^
	Total	≤ 50 (Category A)	51–65 (Category B)	> 65 (Category C)	
Number	8190 (100.0%)	3094 (37.8%)	3703 (45.2%)	1393 (17.0%)	
Age [Years]					
Median	54.0	45.0	58.0	69.0	< 0.0001^b^
Range	19–86	19–50	51–65	66–86	
Study					
SUCCESS A	3754 (45.8%)	1527 (40.7%)	1636 (43.6%)	591 (15.7%)	< 0.0001^a^
SUCCESS B	793 (9.7%)	282 (35.5%)	374 (47.2%)	137 (17.3%)	
SUCCESS C	3643 (44.5%)	1285 (35.3%)	1693 (46.5%)	665 (18.3%)	
Body mass index [kg/m^2^]					
Median	25.53	25.04	26.30	27.0	< 0.0001^b^
Range	14.17–53.91	14.17–53.33	16.46–53.91	15.43–49.01	
Body mass index [kg/m^2^] categories					
< 18.5 (Underweight)	104 (1.3%)	62 (2%)	31 (0.8%)	11 (0.8%)	< 0.0001^a^
18.5–24.99 (Normal)	3643 (44.5%)	1792 (57.9%)	1413 (38.2%)	438 (31.4%)	
25–29.99 (Overweight)	2630 (32.1%)	798 (25.8%)	1270 (34.3%)	562 (40.3%)	
≥ 30.0 (Obese)	1813 (22.1%)	442 (14.3%)	989 (26.7%)	382 (27.4%)	
ECOG at start of first chemotherapy cycle					
0	6229 (76.1%)	2432 (78.6%)	2814 (76.0%)	983 (70.6%)	< 0.0001^a^
1	1584 (19.3%)	531 (17.2%)	722 (19.5%)	331 (23.8%)	
≥ 2	36 (0.4%)	11 (0.4%)	16 (0.4%)	9 (0.6%)	
Unknown	341 (4.2%)	120 (3.9%)	151 (4.1%)	70 (5.0%)	
Menopausal state					
Premenopausal	3295 (40.2%)	2807 (90.7%)	487 (13.2%)	1 (0.1%)	< 0.0001^a^
Postmenopausal	4895 (59.8%)	287 (9.3%)	3216 (86.8%)	1392 (99.9%)	
Tumor stage					
pT1	3546 (43.4%)	1432 (46.3%)	1625 (43.9%)	489 (35.1%)	< 0.0001^a^
pT2	4105 (50.1%)	1516 (49.0%)	1805 (48.7%)	784 (56.3%)	
pT3	402 (4.9%)	113 (3.7%)	205 (5.5%)	84 (6.0%)	
pT4	111 (1.4%)	21 (0.7%)	55 (1.5%)	35 (2.5%)	
Unknown	26 (0.3%)	12 (0.4%)	13 (0.4%)	1 (0.1%)	
Nodal stage					
pN0	3227 (39.4%)	1380 (44.6%)	1413 (38.2%)	434 (31.2%)	< 0.0001^a^
pN1	3667 (44.8%)	1319 (42.6%)	1748 (47.2%)	600 (43.1%)	
pN2	880 (10.7%)	283 (9.1%)	373 (10.1%)	224 (16.1%)	
pN3	383 (4.7%)	96 (3.1%)	155 (4.2%)	132 (9.5%)	
Unknown	33 (0.4%)	16 (0.5%)	14 (0.4%)	3 (0.2%)	
Hormone receptor status					
Negative	2223 (27.1%)	837 (27.1%)	1004 (27.1%)	382 (27.4%)	0.978^a^
Positive	5944 (72.6%)	2247 (72.6%)	2687 (72.6%)	1010 (72.5%)	
Unknown	23 (0.3%)	10 (0.3%)	12 (0.3%)	1 (0.1%)	
Endocrine therapy (hormone receptor-positive tumors only)					
No	2549 (42.9%)	876 (39.0%)	1209 (45.0%)	464 (45.9%)	< 0.001^a^
Yes	3383 (56.9%)	1367 (60.8%)	1471 (54.7%)	545 (54.0%)	
Unknown	12 (0.2%)	4 (0.2%)	7 (0.3%)	1 (0.1%)	
HER2 Status					
Negative	6430 (78.5%)	2422 (78.3%)	2905 (78.4%)	1103 (79.2%)	0.818^a^
Positive	1675 (20.5%)	640 (20.7%)	758 (20.5%)	277 (19.9%)	
Unknown	85 (1.0%)	32 (1.0%)	40 (1.1%)	13 (0.9%)	
HER2-targeted therapy (HER2-positive tumors only)					
No	215 (12.8%)	62 (9.7%)	90 (11.9%)	63 (22.7%)	< 0.001^a^
Yes	1458 (87.0%)	576 (90.0%)	668 (88.1%)	214 (77.3%)	
Unknown	2 (0.1%)	2 (0.3%)	0 (0.0%)	0 (0.0%)	
Histological type					
Invasive ductal	6248 (76.7%)	2460 (79.5%)	2785 (75.2%)	1039 (74.6%)	< 0.0001^a^
Invasive lobular	952 (11.6%)	273 (8.8%)	489 (13.2%)	190 (13.6%)	
Others	930 (11.4%)	351 (11.3%)	416 (11.2%)	163 (11.7%)	
Unknown	24 (0.3%)	10 (0.3%)	13 (0.4%)	1 (0.1%)	
Grading					
G1	418 (5.1%)	144 (4.7%)	211 (5.7%)	63 (4.5%)	0.014^a^
G2	3947 (48.2%)	1442 (46.6%)	1825 (49.3%)	680 (48.8%)	
G3	3795 (46.3%)	1497 (48.4%)	1652 (44.6%)	646 (46.4%)	
Unknown	30 (0.4%)	11 (0.4%)	15 (0.4%)	4 (0.3%)	
Biological subtype					
Luminal A	3369 (41.1%)	1208 (39.0%)	1588 (42.9%)	573 (41.1%)	0.025^a^
Luminal B	1457 (17.8%)	560 (18.1%)	635 (17.1%)	262 (18.8%)	
Her2 type	1675 (20.5%)	640 (20.7%)	758 (20.5%)	277 (19.9%)	
Triple-negative	1601 (19.5%)	652 (21.1%)	681 (18.4%)	268 (19.2%)	
Unknown	88 (1.1%)	34 (1.1%)	41 (1.1%)	13 (0.9%)	
Type of surgery					
Breast-conserving	5852 (71.5%)	2219 (71.7%)	2702 (73.0%)	931 (66.8%)	< 0.0001^a^
Mastectomy	2027 (24.7%)	767 (24.8%)	851 (23.0%)	409 (29.4%)	
Other	290 (3.5%)	99 (3.2%)	139 (3.8%)	52 (3.7%)	
Unknown	21 (0.3%)	9 (0.3%)	11 (0.3%)	1 (0.1%)	
Chemotherapy					
FEC (3x)- > DOC (3x)	4109 (50.2%)	1523 (49.2%)	1881 (50.8%)	705 (50.6%)	0.011^a^
FEC (3x)- > DOC-G (3x)	2254 (27.5%)	916 (29.6%)	985 (26.6%)	353 (25.3%)	
DOC-C (6x)	1827 (22.3%)	655 (21.2%)	837 (22.6%)	335 (24.1%)	
Radiation					
No	1239 (15.1%)	474 (15.3%)	534 (14.4%)	231 (16.6%)	0.158^a^
Yes	6922 (84.5%)	2606 (84.2%)	3155 (85.2%)	1161 (83.3%)	
Unknown	29 (0.4%)	14 (0.5%)	14 (0.4%)	1 (0.1%)	
Dose reduction (cycles 1–6)					
No	7033 (85.9%)	2701 (87.3%)	3198 (86.4%)	1134 (81.4%)	< 0.0001
Yes	1157 (14.1%)	393 (12.7%)	505 (13.6%)	259 (18.6%)	
Dose delay > 6 days due to toxicity (cycle 1–6)					
No	7135 (87.1%)	2715 (87.8%)	3243 (87.6%)	1177 (84.5%)	0.006
Yes	1055 (12.9%)	379 (12.2%)	460 (12.4%)	216 (15.5%)	

^a^Chi-square test

^b^Kruskal–Wallis Test

^§^*p* values calculated without category “unknown”

**Fig. 1:**
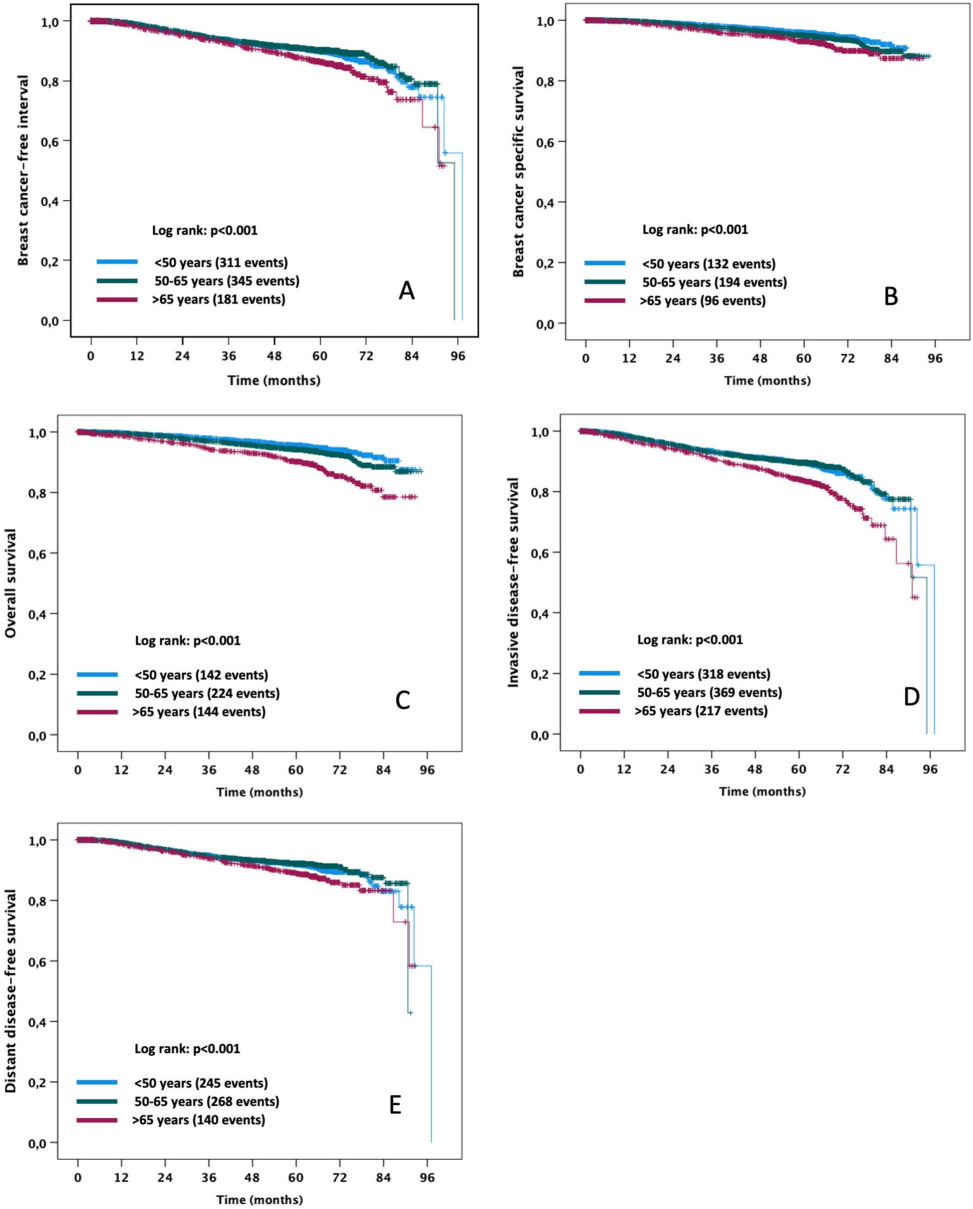
Kaplan–Meier plots of survival according to age category. *p* values refer to global log-rank tests for differences among the three age categories. **A** breast cancer-free interval, **B** breast cancer-specific survival, **C** overall survival, **D** invasive disease-free survival, **E** distant disease-free survival

The median follow-up in months was 64.2 for OS, 63.3 for iDFS, 64.2 for BCSS, and 63.4 for DDFS. Overall, 510 deaths were observed during the study, of which 88 (17.3%) were regarded as not being related to breast cancer. The proportion of deaths that were not related to breast cancer was significantly different among the three age categories (*p < *0.001), with the highest proportion in patients > 65 years (48 out of 144 deaths, 33.3%) compared to patients between 51 und 65 years of age (30 out of 224 deaths, 13.4%) and patients ≤ 50 years (10 out of 142 deaths, 7.0%). The Kaplan–Meier survival plots for all five survival endpoints analyzed according to age category with number of events can be found in Fig. 1, and the results of the corresponding univariable Cox regressions are summarized in Table 2.

**Table 2:** Results of univariable Cox regressions for the effect of age category on different survival endpoints

Survival endpoint	Age category	HR 95%CI	*p*
Breast cancer-free interval	≤ 50	0.74 (0.62–0.89)	0.001^#^
	51–65	0.68 (0.57–0.82)	< 0.0001^#^
	> 65	Reference	< 0.001*
Breast cancer-specific survival	≤ 50	0.60 (0.46–0.77)	< 0.001^#^
	51–65	0.72 (0.57–0.92)	0.009^#^
	> 65	Reference	< 0.001*
Overall survival	≤ 50	0.43 (0.34–0.54)	< 0.0001^#^
	51–65	0.56 (0.45–0.69)	< 0.0001^#^
	> 65	Reference	< 0.0001*
Invasive disease-free survival	≤ 50	0.63 (0.53–0.75)	< 0.0001^#^
	51–65	0.61 (0.52–0.72)	< 0.0001^#^
	> 65	Reference	< 0.0001*
Distant disease-free survival	≤ 50	0.74 (0.60–0.92)	0.005^#^
	51–65	0.68 (0.56–0.84)	< 0.001^#^
	> 65	Reference	0.002*

^*^p value for likelihood ratio test

^#^*p* value for parameter estimation

The global log-rank tests revealed highly significant differences among the three age categories for all five survival endpoints (Fig. 1). Accordingly, univariable Cox regression analysis showed significantly better BCFI, BCSS, OS, iDFS, and DDFS of patients ≤ 50 years of age compared to the patients older than 65 years (reference category), with hazard ratios ranging from 0.43 to 0.74 (all *p < *0.01; see Table 2). Likewise, patients between 51 and 65 years of age also showed significantly better BCFI, BCSS, OS, iDFS, and DDFS compared to patients older than 65 years (hazard ratios ranging from 0.56 to 0.72, all *p < *0.01, Table 2).

The adjusted multivariable Cox regression analysis showed a significant independent age effect only for iDFS (*p = *0.038), but not for the survival endpoint of special interest BCFI nor any of the other survival endpoints investigated (Table 3). Patients between 51 and 65 years had significantly better iDFS than patients older than 65 years (HR 0.80; 95% CI 0.67–0.95; *p = *0.012). However, patients ≤ 50 years of age did not show significantly better iDFS compared to patients older than 65 years in the adjusted analysis (HR 0.88; 95% CI 0.66–1.18; *p = *0.397). After adjusting for other parameters, tumor stage, nodal status, sHR status, and grading remained significant prognostic factors for all survival endpoints, and the use of radiotherapy was a significant prognostic factor in the adjusted multivariable Cox regression analysis for OS, iDFS, and BCFI.

**Table 3:** Results of adjusted multivariable Cox regression models for different survival endpoints

Parameter	Categories	BCFI		BCSS		OS		iDFS		DDFS	
		Hazard Ratio (95% CI)	*p*	Hazard Ratio (95% CI)	*p*	Hazard Ratio (95% CI)	*p*	Hazard Ratio (95% CI)	*p*	Hazard Ratio (95% CI)	*p*
Age			0.286		0.981		0.131		0.038		0.316
	≤ 50 vs. > 65	0.99 (0.74–1.34)	0.954	1.04 (0.68–1.60)	0.844	0.78 (0.53–1.15)	0.214	0.88 (0.66–1.18)	0.397	1.09 (0.78–1.53)	0.621
	51–65 vs. > 65	0.88 (0.73–1.06)	0.171	1.01 (0.78–1.31)	0.936	0.80 (0.64–1.00)	0.047	0.80 (0.67–0.95)	0.012	0.90 (0.73–1.12)	0.340
Study			0.624		0.072		0.026		0.492		0.165
	SUCCESS B vs SUCCESS A	0.85 (0.57–1.25)	0.395	0.84 (0.49–1.43)	0.514	0.90 (0.55–1.48)	0.667	0.87 (0.60–1.27)	0.468	0.69 (0.44–1.07)	0.096
	SUCCESS C vs SUCCESS A	0.94 (0.74–1.20)	0.633	0.68 (0.48–0.96)	0.028	0.65 (0.48–0.89)	0.008	0.89 (0.71–1.13)	0.342	0.88 (0.67–1.16)	0.360
Menopausal status	postmenopausal vs. premenopausal	0.97 (0.75–1.25)	0.808	1.18 (0.82–1.70)	0.378	1.21 (0.86–1.71)	0.282	1.00 (0.78–1.29)	0.983	1.02 (0.77–1.36)	0.887
Body mass index			0.682		0.107		0.067		0.676		0.441
	Normal vs. Underweight	1.11 (0.53–2.36)	0.783	0.61 (0.25–1.50)	0.283	0.60 (0.26–1.35)	0.217	1.03 (0.51–2.08)	0.940	1.15 (0.47–2.80)	0.755
	Overweight vs. Underweight	1.19 (0.56–2.53)	0.656	0.59 (0.24–1.46)	0.252	0.60 (0.26–1.36)	0.216	1.09 (0.54–2.20)	0.818	1.30 (0.53–3.18)	0.561
	Obese vs. Underweight	1.23 (0.58–2.64)	0.588	0.77 (0.31–1.91)	0.576	0.76 (0.33–1.75)	0.523	1.15 (0.56–2.33)	0.709	1.33 (0.54–3.27)	0.532
Type of surgery			0.124		0.224		0.293		0.261		< 0.001
	mastectomy vs. breast-conserving	1.20 (1.00–1.44)	0.045	1.24 (0.97–1.60)	0.085	1.19 (0.95–1.50)	0.128	1.16 (0.97–1.37)	0.102	1.47 (1.21–1.79)	< 0.001
	other vs. breast-conserving	1.16 (0.75–1.79)	0.501	1.15 (0.60–2.23)	0.673	0.95 (0.49–1.82)	0.872	1.07 (0.69–1.65)	0.765	1.08 (0.63–1.84)	0.778
Tumor stage			< 0.001		< 0.001		< 0.001		< 0.001		< 0.001
	pT2 vs. pT1	1.44 (1.23–1.68)	< 0.001	1.91 (1.51–2.42)	< 0.001	1.70 (1.37–2.10)	< 0.001	1.40 (1.20–1.62)	< 0.001	1.52 (1.27–1.82)	< 0.001
	pT3 vs. pT1	1.44 (1.06–1.94)	0.019	2.21 (1.48–3.29)	< 0.001	1.93 (1.33–2.79)	< 0.001	1.43 (1.07–1.92)	0.015	1.23 (0.87–1.75)	0.248
	pT4 vs. pT1	1.96 (1.25–3.09)	0.003	2.44 (1.36–4.39)	0.003	2.29 (1.36–3.84)	0.002	2.01 (1.32–3.08)	0.001	2.11 (1.31–3.42)	0.002
Nodal stage			< 0.001		< 0.001		< 0.001		< 0.001		< 0.001
	pN1 vs. pN0	1.84 (1.53–2.20)	< 0.001	1.99 (1.53–2.59)	< 0.001	1.96 (1.54–2.49)	< 0.001	1.77 (1.48–2.11)	< 0.001	2.07 (1.67–2.55)	< 0.001
	pN2 vs. pN0	3.02 (2.40–3.80)	< 0.001	3.56 (2.59–4.88)	< 0.001	3.45 (2.58–4.62)	< 0.001	2.92 (2.34–3.64)	< 0.001	3.19 (2.45–4.15)	< 0.001
	pN3 vs. pN0	8.06 (6.29–10.32)	< 0.001	9.74 (6.94–13.68)	< 0.001	9.28 (6.78–12.68)	< 0.001	7.69 (6.05–9.77)	< 0.001	8.86 (6.70–11.72)	< 0.001
Hormone receptor status	positive vs. negative	0.38 (0.32–0.46)	< 0.001	0.32 (0.24–0.42)	< 0.001	0.38 (0.29–0.48)	< 0.001	0.42 (0.35–0.50)	< 0.001	0.39 (0.32–0.48)	< 0.001
HER2 status	positive vs. negative	0.69 (0.45–1.05)	0.085	0.75 (0.42–1.35)	0.331	0.91 (0.56–1.50)	0.718	0.84 (0.57–1.25)	0.399	0.67 (0.41–1.10)	0.111
Grading			< 0.001		< 0.001		< 0.001		< 0.001		< 0.001
	G2 vs. G1	2.87 (1.57–5.24)	< 0.001	3.36 (1.24–9.10)	0.017	1.88 (0.96–3.69)	0.065	2.27 (1.35–3.81)	0.002	3.68 (1.73–7.81)	< 0.001
	G3 vs. G1	4.31 (2.35–7.89)	< 0.001	5.38 (1.99–14.56)	< 0.001	2.89 (1.48–5.67)	0.002	3.36 (2.00–5.66)	< 0.001	5.42 (2.55–11.53)	< 0.001
Histology			0.626		0.691		0.900		0.624		0.635
	lobular vs. ductal	0.95 (0.75–1.21)	0.682	1.15 (0.84–1.59)	0.390	1.07 (0.80–1.43)	0.647	0.93 (0.74–1.17)	0.555	0.92 (0.70–1.21)	0.542
	other vs. ductal	0.88 (0.68–1.15)	0.364	1.01 (0.71–1.45)	0.938	1.01 (0.73–1.41)	0.944	0.90 (0.69–1.16)	0.413	0.89 (0.65–1.20)	0.434
Chemotherapy			0.656		0.713		0.927		0.786		0.578
	FEC-DOC-G vs. FEC-DOC	1.00 (0.84–1.19)	0.991	1.02 (0.80–1.30)	0.887	0.98 (0.78–1.22)	0.829	0.97 (0.82–1.15)	0.744	0.98 (0.80–1.19)	0.802
	DOC-C vs. FEC-DOC	1.11 (0.89–1.39)	0.358	1.15 (0.82–1.60)	0.418	1.05 (0.78–1.42)	0.747	1.07 (0.86–1.33)	0.540	1.14 (0.89–1.47)	0.309
ECOG (at start of first chemotherapy cycle)			0.441		0.019		0.017		0.329		0.587
	1 vs. 0	0.95 (0.80–1.12)	0.524	0.93 (0.73–1.18)	0.558	0.96 (0.77–1.19)	0.714	0.95 (0.81–1.12)	0.537	0.92 (0.76–1.12)	0.424
	≥ 2 vs. 0	1.63 (0.67–3.94)	0.281	3.45 (1.41–8.46)	0.007	3.20 (1.42–7.25)	0.005	1.73 (0.77–3.88)	0.185	1.44 (0.46–4.50)	0.535
Endocrine Therapy	yes vs. no	1.11 (0.92–1.34)	0.268	0.83 (0.64–1.09)	0.175	0.75 (0.58–0.96)	0.023	1.03 (0.86–1.23)	0.793	1.07 (0.87–1.32)	0.524
HER2-targeted therapy	yes vs. no	1.09 (0.72–1.66)	0.676	0.82 (0.45–1.49)	0.515	0.64 (0.38–1.07)	0.090	0.88 (0.59–1.31)	0.522	1.22 (0.76–1.95)	0.419
Radiation Therapy	yes vs. no	0.71 (0.56–0.89)	0.003	0.76 (0.55–1.05)	0.095	0.57 (0.44–0.76)	< 0.001	0.61 (0.49–0.75)	< 0.001	1.07 (0.81–1.43)	0.625

*BCFI* breast cancer-free interval, *BCSS* breast cancer-specific survival, *OS* Overall Survival, *iDFS* invasive disease-free survival, *DDFS* distant disease-free survival

**Fig. 2:**
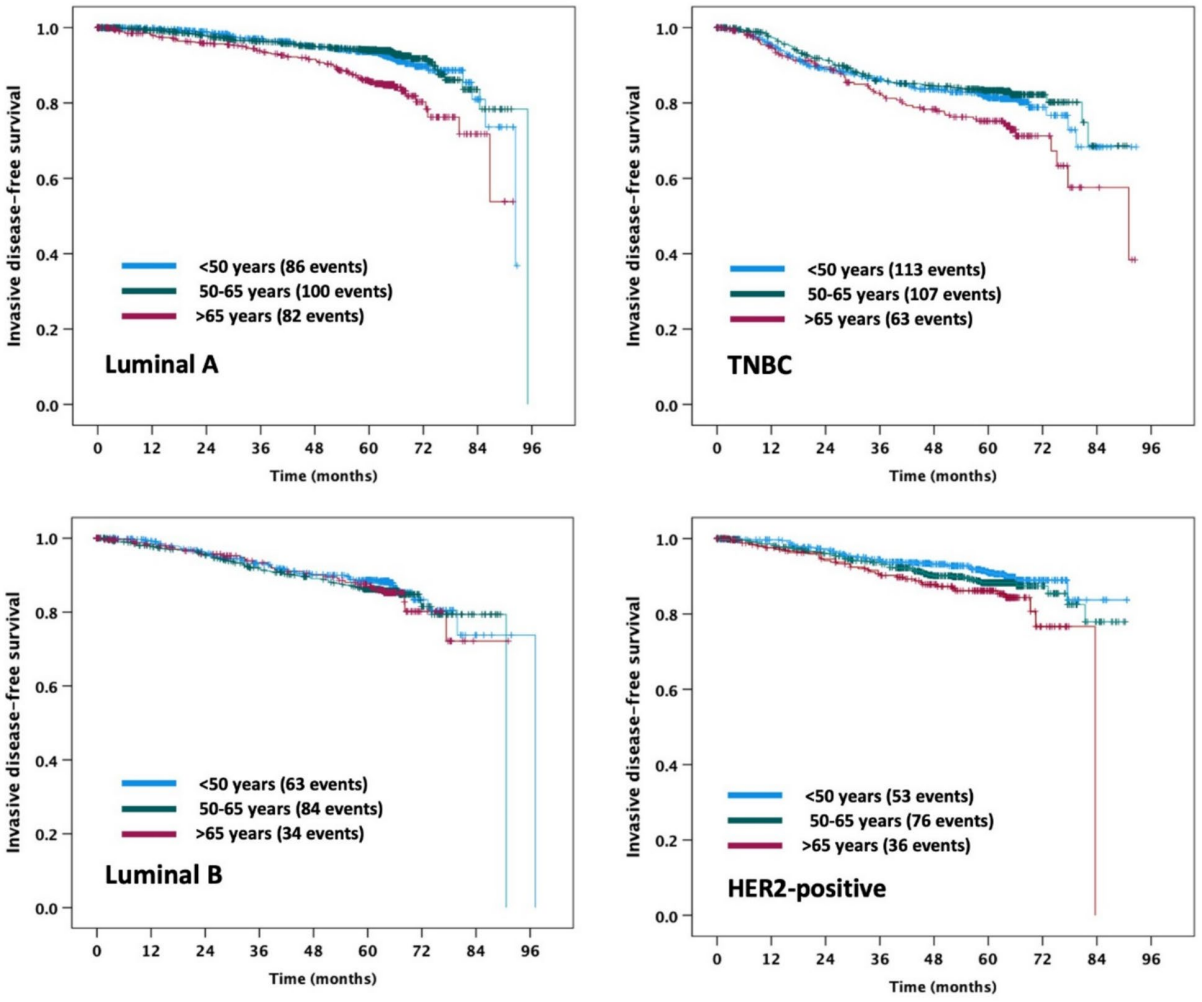
Kaplan–Meier plots for iDFS according to age category and biological subtype. *TNBC* triple-negative breast cancer

**Fig. 3:**
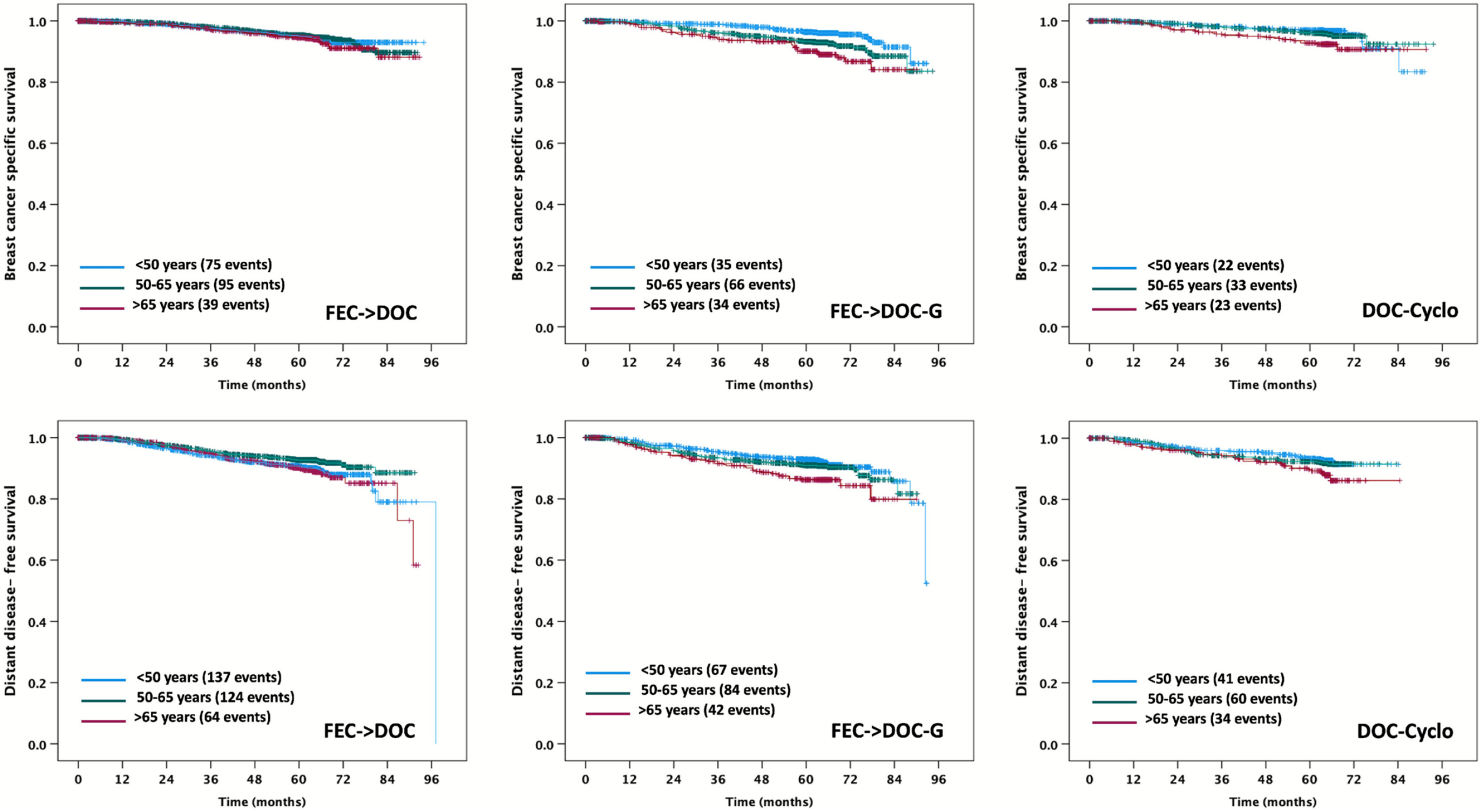
Kaplan–Meier plots for BCSS and DDFS according to age category and type of chemotherapy. FEC-DOC: three cycles of 5-fluorouracil, epirubicin, and cyclophosphamide every 3 weeks followed by 3 cycles of docetaxel q3w. *FEC-DOC-G* Three cycles of 5-fluorouracil, epirubicin, and cyclophosphamide q3w followed by 3 cycles of docetaxel and gemcitabine d1,8 q3w. *DOC-C* Six cycles of docetaxel and cyclophosphamide q3w

For OS, all the tests for 2-way interactions between age category and each of the clinico-pathological parameters investigated (see Table 1) showed no significant results. The same was true for iDFS, with the only exception being a significant 2-way interaction between age category and biological subtype (*p = *0.031), indicating less-pronounced iDFS differences between age categories for patients with luminal B tumors (Fig. 2). The only significant interaction found for BCSS and DDFS (*p = *0.047, *p = *0.044 respectively) was a 2-way interaction between age category and type of adjuvant chemotherapy, suggesting a slightly larger effect of age on these three survival endpoints in patients receiving FEC-DOC-G compared to patients receiving FEC-DOC (Fig. 3).

## Discussion

Data from patients with intermediate- and high-risk eBC participating in one of the three SUCCESS trials were analyzed. The effect of age on survival was evaluated in more than 8000 patients categorized into 3 age groups (≤ 50 years, 51–65 years, > 65 years) that received a standardized adjuvant chemotherapy treatment.

We found significant differences in clinico-pathological parameters among the age groups. On the one hand, our data showed that the proportion of pT3, pT4 tumors, and nodal involvement was higher in patients > 65 years. On the other hand, the proportions of poorly differentiated (G3) carcinomas and triple-negative tumors were the highest in the youngest category. This is consistent with several previously published studies confirming that older patients present a higher proportion of tumors in advanced stages and that with increasing tumor stage, the risk of lymph node involvement also increases [8, 16–19]. In a large study comprising almost 3000 postmenopausal women diagnosed with breast cancer, Gennari et al. reported node positivity rates of 51.1% for patients under the age of 65 and 53.9% for patients ≥ 65 years [20]. This trend toward more lymph node metastases in elderly eBC patients was also evident in our study. Younger patients have been shown to present more frequently with PIK3CA- and BRCA-mutated carcinomas and with increased Ki-67 expression, further emphasizing the more aggressive potential of tumors diagnosed at a younger age [5, 21]. In contrast, older patients with BC have more often sHR -positive tumors, which also present more favorable biological characteristics in terms of normal p53 protein, less frequent HER2- overexpression, and lower grading [8, 22]. It is suggested that limited tissue perfusion and breast involution may be causative for the dominance of slow-growing tumors in older women [23, 24].

In terms of treatment modalities, the mastectomy rate was the highest in the oldest age group, in accordance with previous studies [25]. Even if only 8.5% of patients in this age group had pT3 or pT4 tumors, a mastectomy was performed in 29.4% of the patients. According to the publication of de Boniface et al, breast-conserving surgery with adjuvant radiotherapy showed better OS and BCSS than mastectomy with or without radiation in patients with pT1 and pT2 tumors, regardless of age [26]. Therefore, a higher proportion of patients with a mastectomy in the cohort > 65 years may also have contributed to poorer outcomes in that group. The use of radiotherapy was a significant prognostic factor in the adjusted analysis for OS, iDFS, and BCFI. Additionally, although 72% of the patients in all categories were sHR-positive, only 42% elderly patients (and 49% patients in category A) have endocrine treatment documented. This discrepancy is likely due to endocrine treatment being prescribed by the treating physician rather than selected by the study center, potentially resulting in inadequate documentation of endocrine treatments within the trial. Consequently, reduced compliance with endocrine treatment among elderly study participants in the SUCCESS trials cannot be excluded.

Univariable analysis showed significantly reduced survival for patients older than 65 years for all survival endpoints. These results were obtained despite applying standardized, protocol-based, adjuvant chemotherapy to each patient independent of age. After adjusting for other clinico-pathological factors, we found no significant independent age effect on BCFI, BCSS, OS, and DDFS, a finding that is supported by the results obtained in other studies [27, 28]. Tumor stage, nodal status, sHR status, and grading remained significant in the adjusted analysis, emphasizing the importance of these well-established prognostic factors for guiding treatment decisions in the eBC setting [29–32]. Thus, the deteriorated survival of elderly patients concerning BCFI, BCSS, OS, and DDFS shown in the univariable analysis seems more likely due to advanced tumor stages and higher nodal involvement than to age itself. In this context, it has to be stated that due to the inclusion criterion of an ECOG performance score of ≤ 2 for all three SUCCESS studies, elderly patients > 65 years of age included in the present analysis are likely to be fitter than the average breast cancer patient > 65 years of age, which might partly explain why we did not find an independent age effect regarding OS, DDFS, BCSS, and BCFI in the multivariable adjusted analyses. In contrast to the other survival endpoints, age itself seems to play a role for iDFS in addition to the effects of other known prognostic factors. Why iDFS is worse in the elderly group even if the analysis was adjusted for other prognostic factors is unclear. One possible reason is that iDFS includes non-breast cancer-related deaths as events, which occurred (as to be expected) more often in the elderly patients (see above). Another possible mechanism contributing to this observation could be immunosenescence, where breast cancer cells such as circulating tumor cells shed into the bloodstream more easily evade the immune system of elderly patients and thus are more likely to initiate the metastatic cascade leading to higher recurrence rates [33]. Finally, treatment for elderly patients was more often incomplete due to higher rates of dose reductions or dose delays of more than 6 days because of chemotherapy-induced toxicity observed in our study. This is known to adversely affect outcomes, particularly with regard to anthracyclines [34, 35]. Given that data from the SUCCESS A trial have demonstrated a higher toxicity of FEC-DOC-G as compared to FEC-DOC [28, 36], our results showing similar BCSS, BCFI, and DDFS among patients of the three age categories who had received FEC-DOC, but worse BCSS, BCFI, and DDFS in patients older than 65 years when treated with the more toxic FEC-DOC-G (as confirmed by the significant 2-way interaction effects between age category and type of adjuvant chemotherapy) are in accordance with this suggestion. The reason for poorer survival in older patients receiving usually well-tolerated DOC-C with overall lower toxicity than anthracycline-containing regimens [27, 37] remains unclear—perhaps the higher cumulative taxane and cyclophosphamide dose of DOC-C poses some problems, especially for older patients. However, a separate analysis comprising elderly patients (> 65 years) from the SUCCESS studies with triple-negative breast cancer found no significant differences in survival between patients receiving an anthracycline-containing regimen (FEC-DOC) and patients receiving an anthracycline-free regimen (DOC-C) [38].

The significant 2-way interaction between age category and biological subtype observed for iDFS suggests that patients older than 65 years have worse disease-free survival than younger patients in case of luminal A, HER2-positive or triple-negative tumors, whereas iDFS appears to be more similar across age categories in the case of luminal B tumors. Possible reasons for this finding are unclear. Perhaps under-treatment in older patients is less-pronounced for those with luminal B tumors compared to other tumor subtypes, but such a pattern cannot be easily explained and remains to be demonstrated.

Overall, the effect of age on survival in patients with BC is complex and still not well understood. Previous data have shown that there seem to be no major differences in outcomes in stage-matched patients as age increases [39]. On the other hand, in a large retrospective population-based study, both younger and older age at breast cancer diagnosis were independent risk factors for poorer prognosis after controlling for subtype, lympho-vascular invasion, stage and treatment factors [40]. However, it must be noted that this cohort was different from our study cohort as younger patients were more likely to present with advanced TNM stage at diagnosis and elderly patients were generally more likely to have hormone-sensitive, HER2-negative disease (which was almost balanced in our study population) and lower TNM stage at diagnosis. In many cases, non-guideline-adherent treatment of elderly patients in terms of under-treatment may play an important role [9]. Another issue is the lack of a definite cutoff for age to separate older from younger patients. In different studies, varying cutoffs were used, with younger patients considered as being under 35, 40, or even 50 years, and elderly patients being classified as older than 60, 70, or even 80 years [7, 9, 13, 17, 24]. Furthermore, many effects of age on survival are likely better explained by biological age (as determined by individual health and fitness, comorbidities, etc.) rather than chronological age [41]. Aging-related physiological mechanisms could limit the effect of antineoplastic therapy and consequently negatively affect prognosis in elderly patients.

Besides the mentioned strengths of our study, namely the large number of patients, the clinical trial setting with quality-controlled data acquisition and the standardized adjuvant treatment regimen used, there are also some limiting factors. First, the results presented here were obtained through a retrospective analysis of pooled data from three independent clinical trials that was not planned in advance. Unfortunately, comorbidities and endocrine therapy were not systematically recorded in the study. These are both limitations, as there are naturally implications for survival.

The marginal significance of the 2-way interactions involving age category warrants more data to evaluate both their underlying mechanisms and clinical implications. Finally, the significant age differences among the three SUCCESS trials with their specific interventions, even if accounted for in adjusted multivariable analyses, could cause bias affecting the obtained results, particularly regarding the effects of age × treatment interactions.

## Conclusions

In summary, elderly breast cancer patients over 65 years receiving standardized adjuvant chemotherapy in the SUCCESS trials had poorer survival than younger patients. However, multivariable analysis adjusted for other prognostic factors failed to demonstrate an independent effect of age on BCFI, BCSS, OS, and DDFS, suggesting that the worse survival regarding these four endpoints in patients older than 65 years is more likely attributable to the advanced tumor stages found in the elderly rather than age itself. In contrast to the other survival endpoints, age was shown to be a significant independent prognostic factor for iDFS, indicating an age effect on iDFS in our study population, which however might mainly be attributable to a higher rate of non-breast cancer-related deaths in elderly patients. Due to continuously improving medical care and decreasing mortality at older ages, we can expect increasing incidences of cancer in elderly patients. An effective breast cancer screening strategy in elderly women could help avoid late detection in advanced stages of the disease and reduce the therapy associated morbidity. Finally, more research is needed to fully understand the intrinsic effects of (biological) age on response to cancer therapies.

## Data Availability

No datasets were generated or analyzed during the current study.

## SUCCESS A study synopsis

SUCCESS A was an open-label, multicenter, 2 × 2 factorial design, randomized controlled, Phase III study comparing the disease-free survival after randomization in patients with early primary breast cancer treated with either 3 cycles of Epirubicin–Fluorouracil–Cyclophosphamide (FEC) chemotherapy, followed by 3 cycles of Docetaxel (D) chemotherapy or 3 cycles of Epirubicin–Fluorouracil–Cyclophosphamide (FEC), followed by 3 cycles of Gemcitabine–Docetaxel (DG) chemotherapy, and to compare the disease-free survival after randomization in patients treated with 2 years versus 5 years of adjuvant zoledronate.

Patients were required to have histopathological proof of axillary lymph node metastases (pN1–3) or high-risk node negative, defined as pT ≥ 2 or histopathological grade 3, or age ≤ 35 or negative sHR, but were not allowed to have evidence of distant disease. Patients had to be entered into the study no later than 6 weeks after complete resection of the primary tumor. No other antineoplastic treatment other than surgical treatment, defined cytotoxic, and endocrine treatment and radiotherapy were allowed prior to study entry and during the course of the study. After surgery, leading to R0 resection of the invasive and intra-ductal components of the primary tumor, patients were randomized to one of the following treatments:

### First randomization A

AA: 3 cycles of 5-Fluorouracil 500 mg/m^2^ i.v. body surface area and Epirubicin 100 mg/m^2^ i.v. and Cyclophosphamide 500 mg/m^2^ i.v., (FEC100), each administered on day 1, repeated on day 22, subsequently followed by 3 cycles of Docetaxel 75 mg/m^2^ body surface area i.v. (D), and Gemcitabine 1000 mg/m^2^ i.v. (30 min infusion) (G), administered on day 1, followed by Gemcitabine 1000 mg/m^2^ i.v. (30 min infusion) on day 8, repeated on day 22.

AB: 3 cycles of 5-Fluorouracil 500 mg/m^2^ i.v. body surface area and Epirubicin 100 mg/m^2^ i.v. and Cyclophosphamide 500 mg/m^2^ i.v., (FEC100), each administered on day 1, repeated on day 22, subsequently followed by 3 cycles of Docetaxel 100 mg/m^2^ body surface area i.v. (D), administered on day 1, repeated on day 22.

### Second randomization B

BA: Zoledronic acid 4 mg i.v., every 3 months for the duration of two years, subsequently followed by zoledronic acid 4 mg i.v., every 6 months for the duration of additional three years.

BB: Zoledronic acid 4 mg i.v., every 3 months for the duration of two years.

During the zoledronic acid treatment period, patients received 500 mg Calcium p.o. qid and 400 i.E. Vitamin D p.o. qid.

Patients with positive sHR status (≥ 10% positively stained cells for estrogen and/or progesterone) of the primary tumor received Tamoxifen treatment 20 mg p.o. per day for 2 years after the end of chemotherapy. Postmenopausal patients with positive sHR status were treated with Anastrozole (Arimidex®) 1 mg p.o. for additional 3 years, while premenopausal patients continued Tamoxifen treatment for additional 3 years. In addition to tamoxifen, all patients with positive sHR of the primary tumor and under the age of 40 or restart of menstrual bleeding within 6 months after the completion of cytostatic treatment or with premenopausal hormone levels as defined below received Goserelin (Zoladex®) 3.6 mg subcutaneously every 4 weeks over a period of 2 years following chemotherapy. Premenopausal endocrine status was assumed, if the following serum levels were met: LH < 20 mIE/ml, FSH < 20 mIE/ml and E2 > 20 pg/ml. All patients with breast-conserving therapy or more than 3 axillary lymph node metastases received radiotherapy. In addition, adjuvant radiotherapy was also recommended after mastectomy in case of T3/T4 carcinoma, T2 carcinoma > 3 cm, multi-centric tumor growth, lymphangiosis carcinomatosa, vessel involvement, involvement of the pectoralis fascia or a safety margin < 5 mm.

## SUCCESS B study synopsis

SUCCESS B was an open-label, multicenter randomized controlled, phase III study comparing the disease-free survival after randomization in patients with HER2-neu-positive disease treated with either 3 cycles of Epirubicin–Fluorouracil–Cyclophosphamide (FEC) chemotherapy, followed by 3 cycles of Docetaxel (D) chemotherapy or 3 cycles of Epirubicin–Fluorouracil–Cyclophosphamide (FEC), followed by 3 cycles of Gemcitabine–Docetaxel (DG) chemotherapy.

Patients were required to have HER2-neu-positive disease and histopathological proof of axillary lymph node metastases (pN1-3) or high-risk-node-negative, defined as: pT1c or histopathological grade 2, or age ≤ 35 or negative sHR, if chemotherapy is indicated, but are not allowed to have evidence of distant disease. Patients had to be entered into the study no later than 6 weeks after complete resection of the primary tumor. No other antineoplastic treatment other than surgical treatment, defined cytotoxic, and endocrine treatment and radiotherapy were allowed prior to study entry and during the course of the study.

After surgery, leading to R0 resection of the invasive and intra-ductal components of the primary tumor, patients were randomized to one of the following treatments:

### Randomization

A: 3 cycles of 5-Fluorouracil 500 mg/m^2^ i.v. body surface area and Epirubicin 100 mg/m^2^ i.v. and Cyclophosphamide 500 mg/m^2^ i.v., (FEC100), each administered on day 1, repeated on day 22, subsequently followed by 3 cycles of Docetaxel 75 mg/m^2^ body surface area i.v. (D), and Gemcitabine 1000 mg/m^2^ i.v. (30 min infusion) (G), administered on day 1, followed by Gemcitabine 1000 mg/m^2^ i.v. (30 min infusion) on day 8, repeated on day 22.

B: 3 cycles of 5-Fluorouracil 500 mg/m^2^ i.v. body surface area and Epirubicin 100 mg/m^2^ i.v. and Cyclophosphamide 500 mg/m^2^ i.v., (FEC100), each administered on day 1, repeated on day 22, subsequently followed by 3 cycles of Docetaxel 100 mg/m^2^ body surface area i.v. (D), administered on day 1, repeated on day 22.

After the end of chemotherapy, all patients received biological anti-HER2 treatment with Trastuzumab according to the general therapy guidelines. Postmenopausal patients with positive sHR status (≥ 10% positively stained cells for estrogen and/or progesterone) of the primary tumor received Letrozole treatment 2,5 mg p.o. per day for 5 years, after the end of chemotherapy. Premenopausal patients received Tamoxifen treatment 20 mg p.o. qd. In addition to tamoxifen, all patients with positive sHR status of the primary tumor and under the age of 40 or restart of menstrual bleeding within 6 months after the completion of cytostatic treatment or with premenopausal hormone levels as defined below received Goserelin 3.6 mg subcutaneously every 4 weeks over a period of 2 years following chemotherapy. Premenopausal endocrine status will be assumed, if the following serum levels are met: LH < 20 mIE/ml, FSH < 20 mIE/ml and E2 > 20 pg/ml. All patients with breast-conserving therapy or more than 3 axillary lymph node metastases or in the following cases after mastectomy: T3/T4; R1/R2 Resection; pN + (> 3 involved axillary lymph nodes); in cases of 1–3 involved axillary lymph nodes if additional risk factors are present (lymphangiosis, carcinomatosis, vessel involvement, multi-centric growth, involvement of the pectoralis fascia, and safety margin < 5 mm will receive adjuvant radiotherapy.

## SUCCESS C study synopsis

SUCCESS C was an open-label, multicenter, 2 × 2 factorial design, randomized controlled, phase III study comparing the disease-free survival after randomization in patients treated with either 3 cycles of Epirubicin–Fluorouracil–Cyclophosphamide (FEC) chemotherapy, followed by 3 cycles of Docetaxel (D) chemotherapy, or 6 cycles of Docetaxel– Cyclophosphamide (DC) chemotherapy (first randomization), and to compare the disease-free survival in patients with BMI of 24–40 kg/m^2^ receiving either an intensified lifestyle intervention program or only general lifestyle recommendations (second randomization).

Patients were required to have histopathological proof of a HER2-/neu-negative tumor and axillary lymph node metastases (pN1-3) or high-risk-node-negative, defined as pT ≥ 2 or histopathological grade 3 or age ≤ 35 or negative sHR status, but were not allowed to have evidence of distant disease. Patients had to be entered into the study no later than 6 weeks after complete resection of the primary tumor. No other antineoplastic treatment other than surgical treatment, the defined cytotoxic and endocrine treatment and radiotherapy was allowed prior to study entry and during the course of this study.
